# Associated factors for stroke-related sarcopenia: a scoping review

**DOI:** 10.3389/fneur.2026.1860091

**Published:** 2026-08-04

**Authors:** Fanchi Xu, Yaqin You, Yijing Ling, Yehong Wei, Meng Hu

**Affiliations:** 1 School of Nursing, Zhejiang Chinese Medical University, Hangzhou, China; 2 The Second Affiliated Hospital of Zhejiang Chinese Medical University, Hangzhou, China

**Keywords:** associated factors, rehabilitation, sarcopenia, scoping review, stroke

## Abstract

**Objective:**

To review and summarize research on stroke-related sarcopenia, identify its associated factors, and provide a theoretical foundation for further research and interventions.

**Methods:**

A systematic search was conducted in Chinese National Knowledge Infrastructure (CNKI), Wanfang, VIP, SinoMed, PubMed, Web of Science, CINAHL, Embase, and the Cochrane Library for Chinese and English literature on stroke-related sarcopenia. The search covered publications from the inception of each database through March 4, 2026. The study analyzed and summarized the literature by defining the research topic, screening studies, and conducting methodological evaluations, thereby identifying and summarizing the associated factors associated with stroke-related sarcopenia.

**Results:**

This study included a total of 32 studies, comprising 28 original studies and 4 meta-analyses. Based on the 28 original studies, a total of 32 associated factors for stroke-related sarcopenia were identified, specifically: low BMI, advanced age, low phase angle, reduced calf circumference, low SMI (skeletal muscle index), waist circumference, nutritional status, smoking, low quality of life, decreased activities of daily living, more than three comorbidities, high stroke severity, stroke duration, dysphagia, diabetes, abnormal biochemical markers, tube feeding, length of ICU stay, osteoporosis, number of strokes, cognitive impairment, weight loss, low food texture level, cardiovascular disease, female gender, physical activity level, high risk of falls, oral health issues, pneumonia, speech disorders, gut microbiota diversity, and frailty.

**Conclusion:**

The associated factors for stroke-related sarcopenia are multifaceted. Healthcare professionals need to conduct comprehensive assessments of patients to screen for potential factors associated with stroke-related sarcopenia, thereby providing a basis for clinical intervention.

## 1 Introduction

A stroke is a medical condition defined by the sudden rupture of a blood vessel in the brain, which leads to hemorrhage, or by the blockage of a blood vessel that prevents blood flow to the brain, resulting in ischemia. This condition is marked by a high prevalence, significant mortality rates, and substantial disability (1). Recently, the prevalence and mortality rates associated with stroke have been increasing, making it the second leading cause of death globally (2). As of 2021, there were approximately 93.8 million existing cases of stroke and 11.9 million new cases reported worldwide, creating considerable challenges for stroke treatment and care (3). Projections indicate that over the next 30 years, the number of individuals affected by stroke will rise by 119.83 million, translating to an increase of about 65%, which will substantially elevate the burden on patients, their families, and society as a whole (4).

Following a stroke, due to alterations in neurotransmitters in the target organs of the sympathetic and parasympathetic nervous systems, patients often experience a decline in muscle mass and strength as a result of neurological damage, limb paralysis, limited mobility, and disuse atrophy; At the same time, factors such as dysphagia, inadequate nutritional intake, heightened inflammatory responses, and the long-term burden of rehabilitation may further exacerbate skeletal muscle loss and physical functional decline (5, 6). The combined effect of these multiple factors makes stroke patients more susceptible to sarcopenia, which affects their muscle mass, strength, and physical function, leading to symptoms such as metabolic abnormalities, spasticity, and disuse. This severely impacts patients’ rehabilitation and quality of life. Therefore, sarcopenia has gradually become one of the key concerns in the field of post-stroke rehabilitation. Studies have shown (7) that the prevalence of stroke-related sarcopenia ranges from 16.8 to 60.3%; this variation is associated with factors such as the stage of the disease course (acute phase/chronic phase) and diagnostic criteria (Asian Sarcopenia Working Group, European Working Group on Sarcopenia in Older Adults, International Sarcopenia Working Group, and National Institutes of Health Foundation). However, it is worth noting that existing studies often define stroke-related sarcopenia based on sarcopenic states identified post-stroke using relevant diagnostic criteria or screening tools, rather than on post-stroke-onset sarcopenia confirmed through continuous pre- and post-stroke assessments. Since most studies lack baseline data on pre-stroke muscle mass, muscle strength, or physical function, it is often difficult to strictly distinguish between post-stroke-onset sarcopenia and sarcopenia that existed prior to the stroke but was only identified after the stroke. Therefore, what is referred to as “stroke-related sarcopenia” in current research is, more accurately, a state of sarcopenia identified after the stroke.

Studies have shown (8) that stroke patients with sarcopenia have a 2.71-fold higher risk of adverse outcomes compared to those without sarcopenia, which significantly impacts their prognosis. In addition to physiological effects, a diagnosis of sarcopenia can also have psychological impacts on stroke patients, leading to negative emotions and even symptoms of depression, which hinder their recovery (9). Therefore, early screening for stroke-related sarcopenia and early intervention targeting associated factors are of significant clinical importance for reducing the aforementioned risks and improving the prognosis of stroke patients. Existing research suggests (10) that stroke-related sarcopenia may be associated with multiple factors, including body composition and bone health, stroke severity and comorbidity burden, nutrition and physical function, behavior and environment, and treatment exposure. For example, advanced age, low body mass index (BMI), high stroke severity, smoking, dysphagia, and the presence of other chronic diseases have all been reported to be associated with post-stroke declines in muscle mass and strength. However, the types of factors addressed in current studies are relatively scattered, and the definitions and reporting of these factors vary across studies; a systematic synthesis and analysis are still lacking.

With the update of the expert consensus from the Asian Working Group for Sarcopenia (AWGS), the approach to sarcopenia management is gradually shifting from passive identification to early screening and proactive management. Diagnostic thresholds for sarcopenia in middle-aged adults have been established, marking a transition from a treatment-oriented model to a middle-aged health management model. This involves proactive screening of middle-aged and older adults aged 50–64, while simplifying the diagnostic criteria for sarcopenia to require only the concurrent presence of reduced muscle mass and muscle strength (11). At the same time, clinical assessment and diagnostic methods are continuously being optimized, with screening tools playing a crucial role in the early identification of potential sarcopenia and high-risk conditions. However, existing studies on stroke-related sarcopenia do not fully align in their assessment and diagnostic approaches, ranging from those using diagnostic criteria to those employing screening tools such as the SARC-F. Therefore, this study follows the scoping review framework proposed by Arksey and O’Malley to synthesize the existing evidence on factors associated with stroke-related sarcopenia. It includes relevant studies that use both formal diagnostic criteria and screening tools to present the distribution of research in this field, the main associated factors, and the characteristics of existing studies, thereby providing a reference for the subsequent optimization of screening methods and targeted interventions.

## 2 Materials and methods

### 2.1 Study design

This study followed the scoping review framework proposed by Arksey and O’Malley.

### 2.2 Definition of research questions

Specific review questions: ① What associated factors for stroke-related sarcopenia have been identified in current studies? ② What research designs are predominantly employed in existing studies to investigate the associated factors for stroke-related sarcopenia? ③ Are there any controversies or inconsistencies in the research findings concerning the various associated factors for stroke-related sarcopenia across different studies? ④ In published systematic reviews and meta-analyses, which factors are statistically linked to stroke-related sarcopenia?

Inclusion and exclusion criteria were established according to the PCC principles: Population: ① Age ≥ 18 years ② Meets the diagnostic criteria for stroke as outlined in the Chinese Guidelines for the Diagnosis and Treatment of Acute Ischaemic Stroke 2023 or internationally recognised stroke diagnostic criteria, encompassing both ischaemic and haemorrhagic strokes, with no restrictions on the duration of the disease. ③ Individuals who meet the diagnostic criteria for sarcopenia (e.g., AWGS, EWGSOP2, FNIH, etc.).

*Concept*: ① Sarcopenia is clearly defined (meeting any of the AWGS, EWGSOP, or FNIH criteria, or confirmed by relevant tests, such as the SARC-F brief screening scale) ② The study reports at least one potentially related factor (e.g., body composition and bone health, stroke severity and comorbidity burden, nutrition and physical function, behavioral and environmental factors, treatment factors, etc.).

*Context*: ① There are no restrictions on the type of studies included; this encompasses cohort studies, case–control studies, cross-sectional studies, systematic reviews, and meta-analyses. ② The primary outcomes focus on the prevalence of post-stroke sarcopenia and its associated factors. ③ Articles must be published in either Chinese or English.

*Exclusion criteria*: ① Age < 18 years; ② Non-stroke patients; ③ Studies without clearly defined diagnostic criteria for stroke-related sarcopenia (e.g., AWGS, EWGSOP2, FNIH, etc.); ④ Studies involving sarcopenia diagnosed prior to stroke (e.g., age-related sarcopenia) or non-stroke-related sarcopenia (e.g., kidney disease-related sarcopenia); ⑤ Patients with severe hepatic or renal failure, malignant tumors, or end-stage disease; ⑥ Literature not in Chinese or English; ⑦ Literature for which the full text could not be obtained, duplicate publications, and conference papers; ⑧ Literature published without peer review.

### 2.3 Literature search strategy

Chinese Search Query (using China National Knowledge Infrastructure (CNKI) as an example): Subject: (stroke + cerebral stroke + cerebral infarction + cerebral hemorrhage + cerebrovascular accident) and Subject: (sarcopenia + muscle loss + muscle atrophy + skeletal muscle atrophy + muscle wasting + skeletal muscle reduction).

English Search Query (using PubMed as an example):

**Table:** 

#1 Search: “Stroke”[Mesh]
#2 Search: Stroke OR Cerebrovascular Accident OR Cerebral Stroke OR Stroke, Cerebral OR Cerebrovascular Apoplexy OR Brain Vascular Accident OR Cerebrovascular Stroke OR Stroke, Cerebrovascular OR Apoplexy OR CVA OR Stroke, Acute OR Cerebrovascular Accident, Acute
#3 #1 OR #2
#4 Search: “Sarcopenia”[Mesh]
#5 Search: Sarcopenia OR muscle loss OR muscle reduction OR muscle mass OR muscle atrophy
#6 #4 OR #5
#7 #3 AND #6

### 2.4 Literature screening and analysis

The literature was imported into EndNote software to remove duplicate entries. Literature screening, full-text evaluation, and data extraction were independently conducted by two researchers who had undergone standardized training and were capable of reading both Chinese and English literature. This study included both Chinese and English literature. For Chinese literature, the researchers read the original texts directly to perform screening and data extraction; for English literature, the researchers likewise read the full texts directly to complete screening and extraction, without relying on machine translation as the primary basis for information extraction. After the two researchers completed their independent data extraction, they cross-checked their findings. Any discrepancies: The researchers resolved any discrepancies through discussion, and if they could not reach a consensus through discussion, a third researcher made the final decision. The extracted information included the authors, study type, sample size, number of participants with sarcopenia, prevalence of sarcopenia, diagnostic criteria, and associated factors.

### 2.5 Criteria for assessing study quality

For the cross-sectional studies, cohort studies, case–control studies, and systematic reviews included in this study, the relevant quality assessment tools from the JBI Center for Evidence-Based Healthcare (12) were used. The assessment tool for cross-sectional studies comprised 8 items, with each item rated as “Yes,” “No,” “Unclear,” or “Not Applicable.” The evaluation tool for cohort studies included 11 items, with each item assessed as “Yes,” “No,” “Unclear,” or “Not Applicable.” The evaluation tool for case–control studies included 10 items, with each item assessed as “Yes,” “No,” “Unclear,” or “Not Applicable.” The systematic review and literature evaluation tool consists of 11 items, each with four options: “Yes,” “No,” “Unclear,” and “Not applicable.” This study used the relevant JBI evaluation tools to assess the methodological characteristics of the included literature. This evaluation was primarily used to describe the methodological characteristics underlying the evidence, rather than to exclude studies or conduct a formal determination of evidence grades.

### 2.6 Evidence sources and synthesis strategies

This study included two types of evidence sources: original studies and meta-analyses. Original studies served as the primary evidence base for this study, Which was used to map the distribution of studies, examine study design characteristics, and identify relevant factors. Meta-analyses were primarily used to supplement the relevant factors and their statistical associations previously reported in comprehensive reviews of prior evidence, and to provide a comparative interpretation alongside the results of the original studies. Given that the included meta-analyses may contain primary studies overlapping with those in this study, to avoid double-counting at the conceptual level, this study did not combine meta-analyses and primary studies as equivalent, independent units of evidence for statistical analysis, nor did it include their pooled effect sizes in the cumulative count of relevant factors.

## 3 Results

### 3.1 Results of literature screening

**Figure 1:**
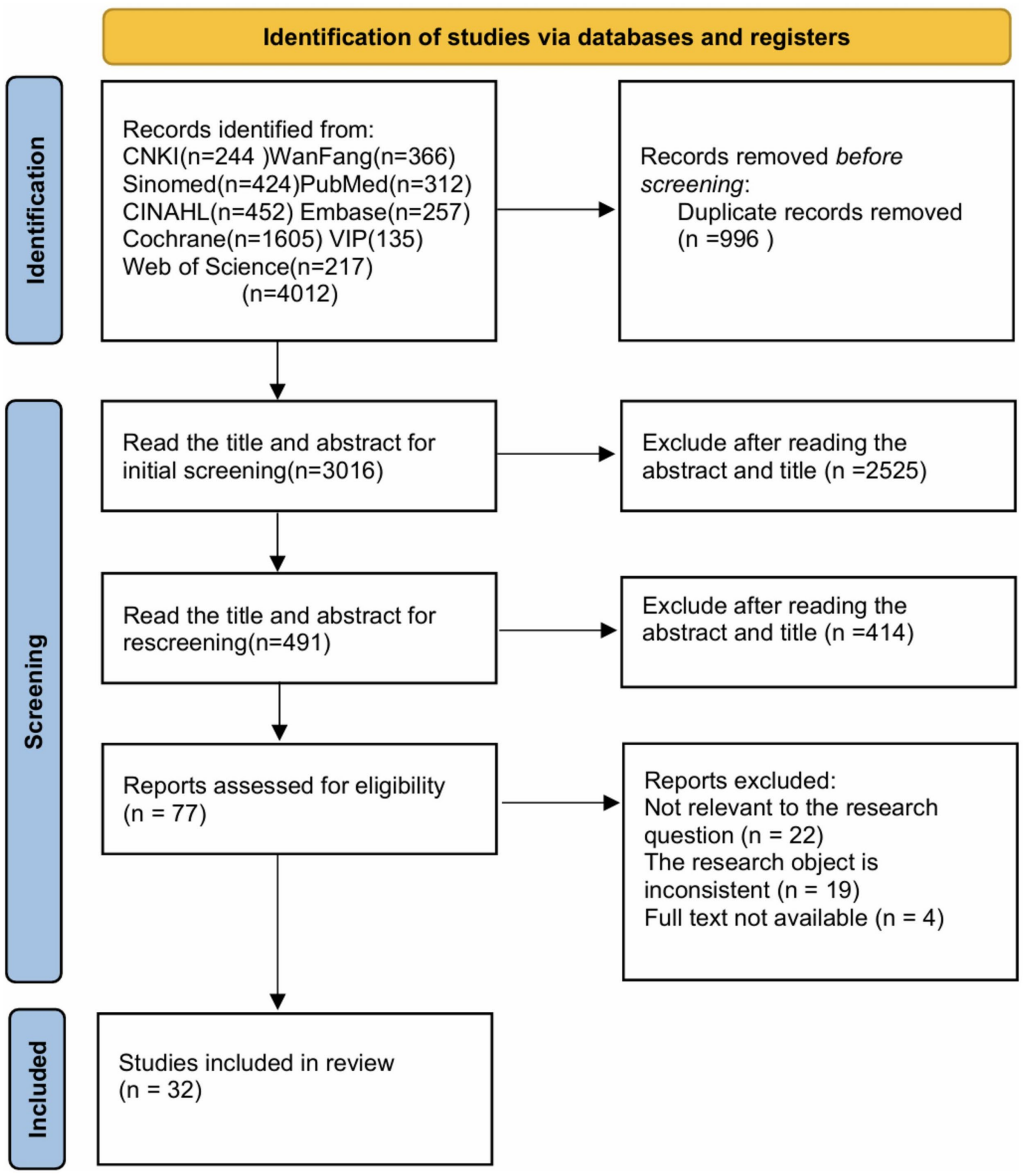
Literature screening flowchart. Adapted from (66).

An initial search identified 4,012 studies. After removing duplicates using EndNote X9, 3,016 studies remained. Following a review of titles, abstracts, and keywords, 77 studies were shortlisted; after a full-text review, 32 studies were ultimately included. The literature screening flowchart is shown in Figure 1.

### 3.2 Basic characteristics of included studies

**Figure 2:**
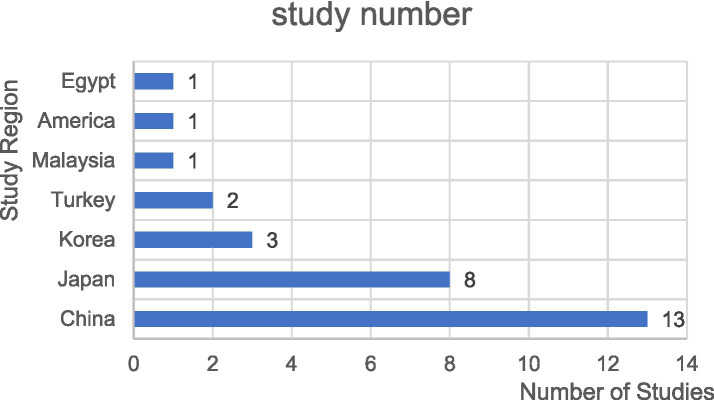
Bar chart of study regions.

**Figure 3:**
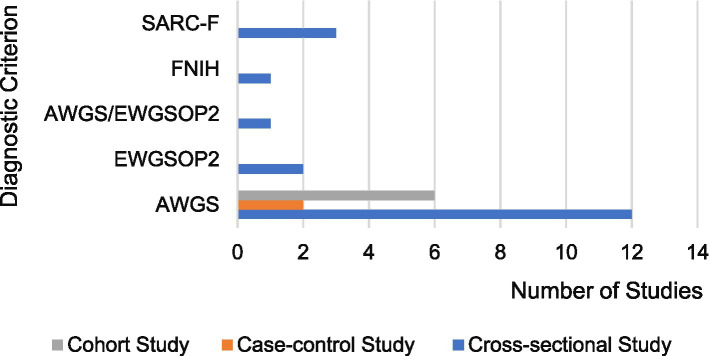
Bar chart of diagnostic criteria and study types.

Among the 32 included studies, 12 were in Chinese and 20 were in English. The basic characteristics of the included studies and their associated factors are shown in Table 1; OR and MD values are presented in Table 2; the classification of associated factors for stroke-related sarcopenia and a summary of meta-analysis evidence are shown in Table 3; bar charts showing the study regions are presented in Figure 2; and bar charts showing diagnostic criteria and study types are presented in Figure 3.

**Table 1:** Basic characteristics and associated factors included in the studies.

Authors	Country	Study type	Sample size	Number of sarcopenia cases	Prevalence of sarcopenia	Diagnostic criteria/screening tools	Associated factors
Yoshimura et al. (39)	Japan	Retrospective cohort study	598	16	46.2%	AWGS	Abnormal biochemical markers
Jang et al. (38)	South Korea	Cross-sectional study	66	12	18.1%	AWGS	Length of ICU Stay
Wang et al. (34)	China	Case–control study	140	70	50%	AWGS	>3 comorbidities, abnormal biochemical markers, osteoporosis, weight loss
Lei et al. (37)	China	Cross-sectional study	111	61	55%	AWGS	Combined Diabetes
Aydin et al. (13)	Turkey	Cross-sectional study	81	26	32.1%	EWGSOP2	Advanced age, duration of stroke
Yoshimura et al. (40)	Japan	Retrospective cohort study	813	385	47.4%	AWGS	Abnormal biochemical markers
Yao et al. (14)	China	Case–control study	259	121	46.7%	AWGS	Advanced age, abnormal biochemical markers, tube feeding, length of ICU stay, cognitive impairment, weight loss, female gender, pneumonia, speech impairment
Shimizu et al. (42)	Japan	Cross-sectional study	443	275	62.1%	EWGSOP2	Nutritional status, low food texture level
Wong et al. (15)	Malaysia	Cross-sectional study	196	83	42.3%	AWGS	Low BMI, advanced age, nutritional status, low quality of life, number of strokes, physical activity
Li et al. (43)	United States	Cross-sectional study	27	5/7	18–25%	AWGS/EWGSOP2	Stroke Duration, Motor Status
Sato et al. (33)	Japan	Cross-sectional study	211	65	30.8%	AWGS	Low phase angle
Chen et al. (16)	China	Cross-sectional study	80	20	25%	AWGS	Low BMI, advanced age, nutritional status, abnormal biochemical markers, osteoporosis, number of strokes
Li et al. (31)	China	Retrospective cohort study	200	100	50%	AWGS	Low BMI, reduced calf circumference, low SMI (skeletal muscle index)
Ikeji et al. (17)	Japan	Prospective cohort study	286	93	32.5%	AWGS	Low BMI, advanced age, high stroke severity, dysphagia
Kim et al. (18)	South Korea	Retrospective cohort study	152	104	68.4%	AWGS	Advanced age, reduced calf circumference, high stroke severity, tube feeding
Cheng et al. (36)	China	Cross-sectional study	187	113	60.43%	SARC-F	High stroke severity, abnormal biochemical markers
Kong et al. (19)	China	Cross-sectional study	489	184	37.6%	SARC-F	Advanced age, nutritional status, smoking, reduced functional independence, physical activity, and high risk of falls
Pu et al. (29)	China	Cross-sectional study	209	33	15.8%	FNIH	Low BMI, waist circumference, abnormal biochemical markers, females
Koca et al. (20)	Turkey	Cross-sectional study	53	28	52.8%	SARC-F	Advanced age, nutritional status, low quality of life, high stroke severity, dysphagia, abnormal biochemical markers, physical activity, high risk of falls
Mohammed et al. (21)	China / Egypt	Cross-sectional study	200/195	26/27	20–34.4%	AWGS	Advanced age, nutritional status, smoking, high stroke severity, concomitant diabetes, abnormal biochemical markers, concomitant cardiovascular disease
Kim et al. (22)	South Korea	Cross-sectional study	224	114	50.9%	AWGS	Low BMI, advanced age, female, physical activity status
Yu et al. (23)	China	Cross-sectional study	242	69	28.5%	AWGS	Advanced age, nutritional status, dysphagia, concomitant diabetes, abnormal biochemical markers, osteoporosis
Shiraishi et al. (41)	Japan	Cross-sectional study	760	288	37.9%	AWGS	Oral Health Issues
Deng et al. (24)	China	Cross-sectional study	666	150	22.5%	AWGS	Low BMI, advanced age, low phase angle, abnormal biochemical markers, and decreased ability to care for oneself
Yoshimura et al. (30)	Japan	Retrospective cohort study	598	240	40.1%	AWGS	Women
Yoshimura et al. (35)	Japan	Cross-sectional study	156	/	/	SMI	Gut Microbiome Diversity
Ye et al. (32)	China	Cross-sectional study	118	47	39.8%	AWGS	Low BMI, frailty
Wang et al. (45)	China	Cross-sectional study	425	145	34.1%	AWGS	Tube feeding, number of strokes, pneumonia

**Table 2:** OR and MD values for associated factors in the meta-analysis.

Associated factors	Statistical measures	He et al. (25)	Yan et al. (26)	Guo et al. (27)	Wang et al. (28)
Age	OR/MD (95% CI)	0.43 (0.06–0.81)*	1.04 (1.02–1.06)	1.05 (1.02–1.08)	1.08 (1.05–1.12)
	*p*-value	/	*p* < 0.0001	*p* = 0.003	*p* < 0.01
Gender	OR/MD (95% CI)	Female: 1.36 (1.09–1.70)*	/	Male: 1.09 (0.84–1.41) Female: 1.34 (0.93–1.93)	Men: 0.91 (0.81–1.03)
	*p*-value	/	/	*p* = 0.53/0.11	*p* = 0.134
BMI	OR/MD (95% CI)	/	0.96 (0.76–1.20)	0.95 (0.83–1.08)	1.13 (0.56–2.26)
	*p*-value	/	*p* = 0.71	*p* = 0.44	*p* = 0.739
Combined cardiovascular disease	OR/MD (95% CI)	/	1.53 (1.15–2.02)	② 0.94 (0.53–1.65)	1.08 (0.86–1.35)
	*p*-value	/	*p* = 0.003	*p* = 0.82	*p* = 0.491
Combined diabetes	OR/MD (95% CI)	/	/	1.16 (0.96–1.39)	1.60 (1.15–2.22)
	*p*-value	/	/	*p* = 0.12	*p* < 0.01
Hyperlipidemia	OR/MD (95% CI)	/	1.56 (1.40–1.74)	/	/
	*p*-value	/	*p* < 0.00001	/	/
Osteoporosis	OR/MD (95% CI)	/	1.80 (1.58–2.04)	/	2.42 (1.95–2.99)
	*p*-value	/	*p* < 0.001	/	*p* < 0.01
Decreased muscle strength	OR/MD (95% CI)	/	/	/	2.04 (1.43–2.90)
	*p*-value	/	/	/	*p* < 0.01
NIHSS score	OR/MD (95% CI)	/	1.48 (1.21–1.81)	1.23 (1.10–1.38)	1.14 (1.10–1.39)
	*p*-value	/	*p* = 0.0001	*p* = 0.00002	*p* < 0.01
History of stroke	OR/MD (95% CI)	/	/	1.33 (1.19–1.49)	1.28 (1.05–1.55)
	*p*-value	/	/	*p* < 0.00001	*p* < 0.01
Lung infection	OR/MD (95% CI)	3.21 (1.98–5.20)*	/	2.90 (0.96–8.73)	1.76 (1.04–2.18)
	*p*-value	/	/	*P* = 0.06	*p* < 0.01
FOIS score	OR/MD (95% CI)	/	0.91 (0.43–1.91)	3.28 (1.83–5.87)	/
	*p*-value	/	*p* = 0.80	*p* < 0.00001	/
Prolonged hospital stay	OR/MD (95% CI)	0.40 (0.20–0.59)*	/	1.01 (0.98–1.04)	1.27 (1.11–1.46)
	*p*-value	/	/	*p* = 0.53	*p* < 0.01
Decreased hemoglobin	OR/MD (95% CI)	−0.47 (−0.62 to −0.32)*	/	0.89 (0.74–1.08)	0.97 (0.94–1.01)
	*p*-value	/	/	*p* = 0.25	*p* = 0.100
Tube feeding	OR/MD (95% CI)	/	3.98 (2.12**–**7.47)	/	2.48 (1.38–4.45)
	*p*-value	/	*p* < 0.0001	/	*p* < 0.01
Smoking history	OR/MD (95% CI)	/	/	/	1.13 (0.56–2.26)
	*p*-value	/	/	/	P = 0.739
Time spent in bed	OR/MD (95% CI)	/	/	0.90 (0.74–1.08)	/
	*p*-value	/	/	*p* = 0.24	/
Comorbidity Index (CCI)	OR/MD (95% CI)	/	/	1.05 (1.02–1.08)	/
	*p*-value	/	/	*p* = 0.00008	/
Stroke duration	OR/MD (95% CI)	0.20 (0.05–0.34)*	0.96 (0.88–1.15)	/	/
	*p*-value	/	*p* = 0.34	/	/
Decreased calf circumference	OR/MD (95% CI)	/	2.36 (0.22–25.70)	/	/
	*p*-value	/	*p* = 0.48	/	/
Sarcopenia before stroke	OR/MD (95% CI)	/	1.84 (1.39–2.43)	/	/
	*p*-value	/	*p* < 0.0001	/	/
Decrease in EQ-5D score	OR/MD (95% CI)	/			0.81 (0.66–1.01)
	*p*-value	/			*p* = 0.063
Decreased albumin	OR/MD (95% CI)	−0.43 (−0.58 to −0.28)*			
	*p*-value	/			

① Atrial fibrillation, ② Atherosclerosis; * indicates MD.

**Table 3:** Classification of associated factors for stroke-related sarcopenia and summary of meta-analysis evidence.

Types of factors in original studies	Number	Proportion (rounded to two decimal places)	Relevant factors in original studies	Factors in the meta-analysis (statistically significant)
Body composition and bone health	10	31.25%	Gender, low BMI, advanced age, low phase angle, reduced calf circumference, low SMI, reduced waist circumference, weight loss, osteoporosis, reduced gut microbiota diversity	Advanced age, gender, pre-stroke sarcopenia, low BMI, osteoporosis
Stroke severity and burden of comorbidities	8	25%	High stroke severity, stroke duration, number of strokes, concomitant diabetes, abnormal biochemical markers, concomitant cardiovascular disease, pneumonia, >3 comorbidities	Stroke duration, pneumonia, concomitant cardiovascular disease, concomitant diabetes, NIHSS score, hyperlipidemia, history of stroke, Comorbidity Index (CCI)
Nutrition and physical function	9	28.13%	Poor nutritional status, low quality of life, decreased ability to care for oneself, dysphagia, cognitive impairment, speech disorders, high risk of falls, oral health problems, frailty	Low hemoglobin, low albumin, FOIS score, decreased muscle strength
Behavior and environment	3	9.38%	Smoking, intake of foods with low texture, physical activity	/
Treatment exposure	2	6.25%	ICU length of stay, tube feeding	Prolonged hospital stay, nasogastric feeding

### 3.3 Results of the literature quality assessment

This study included 20 cross-sectional studies, 2 case–control studies, 6 cohort studies, and 4 systematic reviews. After evaluation, all cross-sectional studies, case–control studies, cohort studies, and systematic reviews were found to have relatively complete study designs and reliable methodology. See Tables 4–7 for details.

**Table 4:** Results of quality assessment for cross-sectional studies.

Included studies	Evaluation criteria								
	①	②	③	④	⑤	⑥	⑦	⑧	Is it included?
Jang et al. (38)	Yes	Yes	Yes	Yes	Yes	Yes	Yes	Yes	Yes
Lei et al. (37)	Yes	Yes	Yes	Yes	No	No	Yes	Yes	Yes
Aydin et al. (13)	Yes	Yes	Yes	Yes	Yes	Yes	Yes	Yes	Yes
Shimizu et al. (42)	Yes	Yes	Yes	Yes	Yes	Yes	Yes	Yes	Yes
Wong et al. (15)	Yes	Yes	Yes	Yes	Yes	Yes	Yes	Yes	Yes
Li et al. (43)	Yes	Yes	Yes	Yes	Yes	Yes	Yes	Yes	Yes
Chen et al. (16)	Yes	Yes	Yes	Yes	Yes	Yes	Yes	Yes	Yes
Cheng et al. (36)	are	Yes	Yes	Yes	Yes	Yes	Yes	Yes	Yes
Kong et al. (19)	Yes	Yes	Yes	Yes	Yes	Yes	Yes	Yes	Yes
Pu et al. (29)	Yes	Yes	Yes	Yes	Yes	Yes	Yes	Yes	Yes
Koca et al. (20)	Yes	Yes	Yes	Yes	Yes	Yes	Yes	Yes	Yes
Mohammed et al. (21)	Yes	Yes	Yes	Yes	Yes	Yes	Yes	Yes	Yes
Kim et al. (22)	Yes	Yes	Yes	Yes	Yes	Yes	Yes	Yes	Yes
Yu et al. (23)	Yes	Yes	Yes	Yes	Yes	Yes	Yes	Yes	Yes
Shiraishi et al. (41)	Yes	Yes	Yes	Yes	Yes	Yes	Yes	Yes	Yes
Deng et al. (24)	Yes	Yes	Yes	Yes	Yes	Yes	Yes	Yes	Yes
Yoshimura et al. (30)	Yes	Yes	Yes	Yes	Yes	Yes	Yes	Yes	Yes
Sato et al. (33)	Yes	Yes	Yes	Yes	Yes	Yes	Yes	Yes	Yes
Ye et al. (32)	Yes	Yes	Yes	Yes	Yes	Yes	Yes	Yes	Yes
Wang et al. (45)	Yes	Yes	Yes	Yes	Yes	Yes	Yes	Yes	Yes

① Were the inclusion criteria for study participants clearly defined? ② Were the study participants and study sites described in detail? ③ Were valid and reliable methods used to assess exposure factors? ④ Were objective and standardized methods used to assess health problems? ⑤ Were confounding factors identified? ⑥ Were measures taken to control for confounding factors? ⑦ Were valid and reliable methods used to assess outcome measures? ⑧ Were appropriate data analysis methods used?

**Table 5:** Results of the quality assessment of case–control studies.

Included studies	Evaluation criteria										
	①	②	③	④	⑤	⑥	⑦	⑧	⑨	⑩	Is it included
Wang et al. (34)	Yes	Yes	Yes	Yes	Yes	Yes	Yes	Yes	Yes	Yes	Yes
Yao et al. (14)	Yes	No	Yes	Yes	Yes	Yes	Yes	Yes	Yes	Yes	Yes

① Aside from the fact that the disease is present in the case group but absent in the control group, are the groups comparable? ② Are cases and controls appropriately matched? ③ Were the same criteria used to identify cases and controls? ④ Was exposure measured in a standardized, valid, and reliable manner? ⑤ Were exposure measurements conducted in the same way for both the case and control groups? ⑥ Were confounding factors identified? ⑦ Were strategies for addressing confounding factors described? ⑧ Were the results evaluated in a standardized, valid, and reliable manner for both the case and control groups? ⑨ Was the exposure period sufficiently long and clinically meaningful? ⑩ Were appropriate statistical analysis methods used?

**Table 6:** Quality assessment of cohort studies.

Included studies	Evaluation criteria											
	①	②	③	④	⑤	⑥	⑦	⑧	⑨	⑩	⑪	Is it included
Yoshimura et al. (30)	Yes	Yes	Yes	Yes	Yes	Yes	Yes	Yes	Yes	Yes	Yes	Yes
Yoshimura et al. (35)	Yes	Yes	Yes	Yes	Yes	Yes	Yes	Yes	Yes	No	Yes	Yes
Li et al. (43)	Yes	Yes	Yes	Yes	Yes	Yes	Yes	Yes	Yes	No	No	Yes
Ikeji et al. (17)	Yes	Yes	Yes	Yes	Yes	Yes	Yes	Yes	Yes	No	No	Yes
Kim et al. (18)	Yes	Yes	Yes	Yes	Yes	Yes	Yes	Yes	No	No	Yes	Yes
Yoshimura et al. (40)	Yes	Yes	Yes	Yes	Yes	Yes	Yes	Yes	No	No	Yes	Yes

① Are the two groups similar and drawn from the same population? ② Are the methods for measuring exposure similar for the exposed and unexposed groups? ③ Was exposure measured in a valid and reliable manner? ④ Were confounding factors identified? ⑤ Were strategies for addressing confounding factors described? ⑥ At the start of the study (or at the time of exposure), were the groups/participants free from bias regarding the study outcomes? ⑦ Were the outcomes measured in a valid and reliable manner? ⑧ Was the follow-up duration reported, and was it long enough to observe the relevant outcomes? ⑨ Was follow-up completed? If not, were the reasons for non-follow-up described and investigated? ⑩ Were strategies implemented to address incomplete follow-up? ⑪ Were appropriate statistical analyses used?

**Table 7:** Results of the systematic review quality assessment.

Included studies	Assessment criteria											
	①	②	③	④	⑤	⑥	⑦	⑧	⑨	⑩	⑪	Is it included
Yan et al. (26)	Yes	Yes	Yes	No	Yes	Yes	Yes	Yes	Yes	Yes	Yes	Yes
He et al. (25)	Yes	Yes	Yes	No	Yes	Yes	Yes	Yes	Yes	Yes	Yes	Yes
Guo et al. (27)	Yes	Yes	Yes	No	Yes	Yes	Yes	Yes	Yes	No	No	Yes
Wang et al. (45)	Yes	Yes	Yes	Yes	Yes	Yes	Yes	Yes	Yes	Yes	Yes	Yes

① Was a preliminary design protocol provided? ② Are the selection of included studies and data extraction reproducible? ③ Was a comprehensive literature search conducted? ④ Were publication-related factors, such as gray literature, considered in the inclusion criteria? ⑤ Is a list of included and excluded studies provided? ⑥ Are the characteristics of the included studies described? ⑦ Was the scientific quality of the included studies evaluated and reported? ⑧ Was the scientific rigor of the included studies appropriately applied in deriving the conclusions? ⑨ Were the methods used to synthesize the results of the included studies appropriate? ⑩ Was the possibility of publication bias assessed? ⑪ Were relevant conflicts of interest disclosed?

### 3.4 Associated factors for stroke-related sarcopenia

This study included a total of 32 studies, comprising 28 original studies and 4 systematic reviews; excluding the systematic reviews, a total of 8,185 stroke patients were included. Among the 28 original studies, 20 used the AWGS as the diagnostic criterion for sarcopenia, 2 used the EWGSOP2, 1 used the FNIH, 1 used a combination of AWGS and EWGSOP2, and 1 used SMI as a screening indicator for sarcopenia, while the remaining three studies used the SARC-F questionnaire as a screening tool for sarcopenia. The studies found that the prevalence of sarcopenia after stroke ranged from 15.8 to 68.4%, which is similar to the results reported by Su et al. (7) (16.8–60.3%). The wide range in prevalence may be related to differences in stroke duration and sample characteristics, as well as to the diagnostic criteria and screening tools used for sarcopenia. This study conducted a systematic quality assessment of the included studies using the JBI evaluation tool. The included studies demonstrated a certain degree of assessability in terms of study design and reporting; however, differences still existed regarding control of confounding factors, follow-up completeness, and measurement consistency. Therefore, the relevant results should be interpreted with caution, taking study heterogeneity into account. Based on this, 32 associated factors for stroke-associated sarcopenia were identified and categorized into five areas: body composition and skeletal health; stroke severity and comorbidity burden; nutrition and physical function; behavior and environment; and treatment exposure.

#### 3.4.1 Body composition and bone health

This study identified the following 10 factors related to body composition and skeletal health from the original studies: gender, low BMI, advanced age, low phase angle, reduced calf circumference, low SMI, reduced waist circumference, weight loss, osteoporosis, and reduced gut microbiota diversity. These can be further subdivided into three categories: demographic factors, muscle and skeletal parameters, and gut microbiota.

##### 3.4.1.1 Demographic factors

Demographic factors primarily include advanced age and gender. Twelve original studies (13–16, 17–24), and 4 meta-analyses (25–28) all consistently indicate that advanced age is a associated factor for stroke-related sarcopenia, likely due to age-related declines in muscle mass, muscle strength, and neural function (26). Four original studies (14, 22, 29, 30) and one meta-analysis (25) indicate that the prevalence of sarcopenia is higher in female stroke patients than in male stroke patients. This may be related to factors such as differences in body composition and changes in hormone levels after menopause (25).

##### 3.4.1.2 Muscle and skeletal-related parameters

This study identified seven factors associated with musculoskeletal parameters: low BMI, low SMI, reduced calf circumference, reduced waist circumference, low phase angle, osteoporosis, and weight loss.

Eight original studies (15–17, 22, 24, 29, 31, 32) and one meta-analysis (28) found that low BMI is an associated factor for stroke-related sarcopenia. Additionally, as one of the core diagnostic indicators of sarcopenia, the skeletal muscle index (SMI) should be given due attention following a stroke (31). Two original studies (18, 31) showed a negative correlation between reduced calf circumference and sarcopenia at discharge. In addition to calf circumference, one original study (29) demonstrated an association between reduced waist circumference and stroke-related sarcopenia. Two original studies (24, 33) found that the phase angle was negatively correlated with post-stroke sarcopenia. The phase angle is a measure derived from bioimpedance analysis that reflects muscle condition and overall nutritional status. Three original studies (16, 23, 34) and two meta-analyses (26, 28) indicate that osteoporosis may be associated with stroke-related sarcopenia. Yan et al. (26) found a combined odds ratio (OR) for osteoporosis of 1.80 (1.58–2.04), while Wang et al. (28) reported an even stronger combined effect of osteoporosis as an associated factor, with a combined OR of 2.42 (1.95–2.99), suggesting a relatively robust statistical association between osteoporosis and stroke-related sarcopenia. Additionally, two studies (14, 34) found that patients with a history of weight loss have a higher risk of osteoporosis and sarcopenia. Indicators such as BMI, body weight, calf circumference, SMI, and phase angle can reflect the body composition, nutritional reserves, and muscle health of stroke patients from different perspectives. Patients with stroke-related functional impairments often have reduced physical activity and restricted food intake; under these circumstances, abnormalities in the indicators mentioned may collectively be associated with the occurrence of sarcopenia.

##### 3.4.1.3 Gut microbiome

Yoshimura et al. (35) found that gut microbiome diversity may play a significant role in post-stroke muscle health. Stroke patients with greater gut microbiome diversity generally exhibit better muscle mass. The gut microbiota contributes to muscle health by regulating systemic inflammatory responses and improving absorption and metabolic functions. A decrease in gut microbiota diversity can negatively influence dietary intake and body mass index (BMI) in stroke patients, which subsequently affects their muscle function. This impact typically becomes evident during the post-stroke rehabilitation period.

#### 3.4.2 Stroke severity and burden of comorbidities

This study identified eight factors associated with stroke severity and the burden of comorbidities, namely high stroke severity, duration of stroke, number of strokes, concomitant diabetes, abnormal biochemical markers, concomitant cardiovascular disease, pneumonia, and having more than three comorbidities.

Five original studies (17, 18, 20, 21, 36), along with three meta-analyses (26–28), all found that high stroke severity is an associated factor for sarcopenia in stroke patients. Stroke severity and duration were assessed using the National Institutes of Health Stroke Scale (NIHSS), which consists of 15 items totaling points; a higher score indicates a more severe stroke (17). One original study (13) and one meta-analysis showed that stroke duration influences the incidence of sarcopenia, with a mean difference (MD) of 0.20 (0.05–0.34). Two original studies (15, 16) found that the number of strokes is a relevant factor; two meta-analyses (27, 28) further explored this factor, with pooled odds ratios (ORs) of 1.28 (1.05–1.55) and 1.33 (1.19–1.49), respectively.

In addition to the severity of the stroke itself, there is also an association between the burden of comorbidities and post-stroke sarcopenia. A study (21) found that in an Egyptian population, a history of diabetes, cardiovascular disease, and dyslipidemia was associated with a 19.8, 19.2, and 21.3% decrease in corresponding muscle strength, respectively. This study also found that the presence of diabetes, cardiovascular disease, and dyslipidemia in stroke patients is an associated factor for sarcopenia. Among these, three original studies (21, 23, 37) found that the presence of diabetes was an associated factor for sarcopenia in stroke patients; a meta-analysis subsequently determined its odds ratio (OR) to be 1.60 (1.15–2.22). Dyslipidemia, on the other hand, leads to fat accumulation, impairs neurological recovery in stroke patients, and increases the risk of sarcopenia, with an OR of 1.56 (1.40–1.74) (27). When patients have more than three comorbidities, the risk of sarcopenia increases significantly (38). These comorbidities may collectively affect muscle mass and function in stroke patients through mechanisms such as chronic inflammation, metabolic abnormalities, reduced physical activity, and increased disease burden.

Eleven original studies (14, 16, 20, 21, 23, 24, 29, 34, 36, 39, 40) and others have found that abnormalities in biochemical markers may influence the development of post-stroke sarcopenia. Among these, C-reactive protein (36, 41) and lymphocyte count (20), as markers of inflammation, can accelerate protein breakdown and inhibit protein synthesis by activating various signaling pathways, potentially influencing the onset and progression of sarcopenia. Three original studies (14, 16, 24) indicate that lower triglyceride levels are associated with stroke-related sarcopenia, whereas higher triglyceride levels act as a protective factor against sarcopenia. Furthermore, two original studies (14, 29) and one meta-analysis (25) found that hemoglobin levels influence the development of sarcopenia, with an MD of −0.47 (−0.62 to −0.32). Additionally, three original studies (14, 29, 34) found that abnormalities in albumin, creatinine, creatine, total calcium, and hormone levels all increase the risk of post-stroke sarcopenia. Among these, decreased albumin—a significant associated factor—was also reported in two meta-analyses (25, 28): MD = −0.43 (−0.58 to 0.28) (25) and OR = 4.27 (2.62 to 6.97) (28). Study (40) further indicated that estimated glomerular filtration rate (eGFR) is associated with sarcopenia and is linked to prognostic factors such as dysphagia, activities of daily living (ADL), and prolonged hospital stay. These biochemical abnormalities may reflect the systemic status of stroke patients at various levels—including inflammation, nutrition, and metabolism—and may influence their muscle mass, strength and, and physical function.

#### 3.4.3 Nutrition and physical function

This study identified a total of 9 factors related to nutrition and physical function, namely: nutritional status, low quality of life, reduced ability to perform activities of daily living, dysphagia, cognitive impairment, speech impairment, high risk of falls, oral health problems, and frailty.

Eight original studies (15, 16, 19–21, 23, 42) found that nutritional imbalance affects muscle mass and strength in stroke patients and also reduces their quality of life. In contrast, optimal nutritional status and early rehabilitation can reduce the incidence of sarcopenia. Among these, a study by Kong et al. (19) indicated that in individuals under 75 years of age, the risk of sarcopenia is 18% when there are moderate to severe impairments in Activities of Daily Living (ADL) and nutritional status is normal; however, this risk rises to 79% when nutritional status is abnormal. Dysphagia may also potentially affect nutritional status and further exacerbate nutritional risks by impairing the ability to eat. One original study (17) and one meta-analysis (27) found an association between dysphagia and stroke-related sarcopenia. Swallowing function can be assessed using the Functional Oral Intake Scale (FOIS), which consists of seven levels; a lower level indicates more severe dysphagia (17). A study (27) found that the odds ratio (OR) for the FOIS score was 3.28 (1.83, 5.87), further illustrating the impact of swallowing dysfunction on nutrition and stroke-related sarcopenia. In addition to the relationship between swallowing dysfunction and nutritional status, four original studies (15, 19, 20, 24)found that reduced functional independence and poor quality of life increase the risk of post-stroke sarcopenia. Functional independence was assessed using the Barthel Index and ADL scores; a decline in functional independence leads to impaired limb mobility, which in turn increases the risk of sarcopenia. Quality of life can be assessed using the EQ-5D questionnaire. A study by Wong et al. (15) found that lower EQ-5D scores may increase the risk of post-stroke sarcopenia in the 60–69 age group. Additionally, two original studies (19, 20) found that a high risk of falls can lead to a fear of physical activity among patients, which in turn reduces physical activity and increases the risk of sarcopenia. Furthermore, post-stroke cognitive impairments and speech disorders, when present, collectively increase patients’ risk of sarcopenia (14). A study (32) further revealed a dose–response relationship between frailty and the incidence of stroke-related sarcopenia, establishing frailty as an independent associated factor for this condition. Factors such as nutritional risk, dysphagia, poor quality of life, and frailty primarily reflect stroke patients’ ability to obtain adequate nutrition, their daily functional status, and their overall condition. Existing research suggests that malnutrition and dysphagia may interact to jointly affect muscle protein synthesis, activity levels, and rehabilitation capacity, thereby influencing the muscle condition of stroke patients.

#### 3.4.4 Behavior and environment

This study identified a total of three behavioral and environmental factors: smoking, low food texture levels, and physical activity. One original study (42) found that stroke patients who consistently consume foods with a softer texture according to the IDDSI classification may have a higher risk of malnutrition and sarcopenia. Two original studies (19, 21) found that smoking is an associated factor for stroke-related sarcopenia. Smoking is a major associated factor for periodontitis and chronic obstructive pulmonary disease (COPD). Both of these conditions can lead to a diminished sense of taste and reduced chewing ability, which in turn can cause swallowing difficulties and decreased physical activity, thereby indirectly affecting nutritional intake and muscle health. In addition, smoking itself may adversely affect skeletal muscle metabolism through mechanisms such as chronic inflammation and oxidative stress. Five studies (15, 43, 19, 20, 22) found that the prevalence of sarcopenia in older stroke patients is associated with their physical activity levels; older stroke patients who exercise regularly have a relatively lower prevalence of sarcopenia. Stroke patients with insufficient physical activity typically exhibit reduced muscle use and inadequate participation in rehabilitation training, thereby increasing the likelihood of post-stroke declines in muscle mass and strength.

In summary, behavioral and environmental factors primarily reflect the lifestyle, dietary habits, and physical activity patterns of post-stroke patients. Existing research suggests that poor behavioral habits and low activity levels may affect muscle status in stroke patients by influencing nutritional intake, inflammatory status, and the extent of muscle use.

#### 3.4.5 Treatment factors

This study identified two treatment-related factors: length of ICU stay and tube feeding. Two original studies (14, 38) and two meta-analyses found that length of ICU stay is associated with the onset of sarcopenia; this association was also reported in two additional meta-analyses (25, 28). A prolonged ICU stay typically indicates more severe illness, longer periods of bed rest, and a higher risk of complications, all of which may adversely affect skeletal muscle mass and function.

Furthermore, patients treated in the ICU often require tube feeding due to severe illness, impaired swallowing function, or altered levels of consciousness. Two original studies (14, 18) have shown that tube feeding is associated with post-stroke sarcopenia. Yan et al. (26) found that tube feeding was significantly associated with the development of post-stroke sarcopenia (OR = 3.98, 2.12–7.47). However, patients receiving tube feeding may also have severe swallowing disorders, high nutritional risk, prolonged immobilization, or severe illness. Therefore, treatment-related factors are inextricably linked to patients’ underlying conditions, and these various factors interact with and influence one another, collectively affecting the development of sarcopenia in stroke patients.

## 4 Discussion

This study reviewed the existing evidence on factors associated with stroke-related sarcopenia and, in conjunction with previous systematic reviews, provided supplementary comparisons of the factors reported in those studies. The results indicate that the associated factors for post-stroke sarcopenia are multifaceted, but various factors may interact with one another, collectively influencing the muscle status of stroke patients. Furthermore, these factors can guide early screening and intervention for sarcopenia risk in stroke patients. Therefore, in clinical practice, healthcare professionals need to provide targeted care based on the specific circumstances of different patient groups, comprehensively assess the physical condition of stroke patients, identify potential and existing associated factors, and develop personalized intervention plans.

### 4.1 Body composition and bone health: key associated factors for stroke-related sarcopenia

Regarding body composition and skeletal health, both original studies and meta-analyses have reported four associated factors: advanced age, gender, low BMI, and osteoporosis. These factors collectively reflect stroke patients’ baseline physical reserves, aging status, and levels of skeletal and muscular health, may be closely associated with the onset of post-stroke sarcopenia.

Several original studies (13–22, 24, 30, 41) and four meta-analyses (25–28) all agree that advanced age is a associated factor for stroke-related sarcopenia, which may be related to the physiological changes associated with ageing. First, middle-aged and older adults are at high risk for stroke. With advancing age, skeletal muscle mass, muscle strength, and neuromuscular function gradually decline, and protein synthesis capacity weakens. Furthermore, post-stroke patients may experience a further decline in physical function due to factors such as limited mobility, inflammatory responses, and reduced nutritional intake (44). Second, the risk of osteoporosis in older adults also increases with age. As a key factor associated with stroke-related sarcopenia, osteoporosis may be more pronounced in women. This may be because postmenopausal women experience reduced oestrogen secretion, leading to accelerated bone loss accompanied by a decline in muscle mass and function, thereby increasing the likelihood of sarcopenia in women following a stroke (22). A study (45) found that a low BMI may influence the development of stroke-related sarcopenia by increasing nutritional risk, whereas a higher BMI indicates that patients have greater metabolic reserves, enabling them to resist the decline in muscle mass and strength caused by excessive catabolism following a stroke. This is consistent with the manifestation of the “obesity paradox” in stroke patients, whereby stroke patients with higher BMIs have better prognoses (46). However, a higher BMI is not necessarily better. Boettger et al. (47), using a restricted cubic spline model, found that BMI in patients with acute ischemic stroke and transient ischemic attacks exhibited a “U-shaped” association with stroke severity and functional outcomes: overweight patients (BMI 25.0–29.9 kg/m^2^) had the lowest NIHSS scores, while lower BMI and obesity were associated with more severe stroke and poorer outcomes. This study indicates a dose–response relationship between BMI and both the severity and functional outcomes of stroke. In contrast, Ye et al. (32) found an “L-shaped” association between BMI and sarcopenia in patients with acute ischemic stroke; where, when BMI < 23.1 kg/m^2^, the risk of stroke-related sarcopenia increases as BMI decreases; when BMI > 23.1 kg/m^2^, the risk of sarcopenia tends to stabilize. This further reveals a dose–response relationship between the occurrence of sarcopenia in stroke patients and their BMI. This dose–response relationship provides valuable guidance for the management of high-risk patients.

In summary, advanced age, female gender, low BMI, and osteoporosis are not isolated associated factors; together, they may reflect insufficient baseline physical reserves, musculoskeletal degeneration, and an increased risk of malnutrition and in stroke patients. Therefore, in the screening for stroke-related sarcopenia, in addition to routine functional assessments, factors such as the patient’s age, body composition, and bone health should also be taken into account.

### 4.2 Stroke severity and burden of comorbidities: key contextual factors in the onset and progression of stroke-related sarcopenia

Stroke severity and the burden of comorbidities together constitute important contextual factors for stroke-related sarcopenia. Elderly stroke patients often present with “multiple coexisting conditions,” such as diabetes, hyperlipidemia, and hypertension. These underlying conditions not only increase the risk of stroke, but also may affect muscle mass, strength, and physical function in stroke patients post-stroke through various mechanisms, including inflammatory responses, metabolic abnormalities, and limited mobility.

Diabetes is one of the comorbidities warranting particular attention. As a major associated factor for cardiovascular disease, chronic hyperglycemia impairs vascular endothelial function, promotes atherosclerosis, and is accompanied by metabolic issues such as hypertension and dyslipidemia; the earlier the onset of type 2 diabetes and the longer the duration of the disease, the higher the risk of stroke (48); simultaneously, stroke patients with diabetes also have a higher risk of sarcopenia (41). The causes may be related to insulin resistance, chronic low-grade inflammation, and abnormalities in protein metabolism (41). Therefore, for stroke patients with diabetes, special attention must be paid to blood glucose levels and other biochemical indicators related to cardiovascular health and metabolic status to enable early detection of changes in the patient’s condition and timely intervention.

Furthermore, factors such as high stroke severity, a higher NIHSS score, and longer stroke duration all indicate a more severe condition. Patients with more severe conditions tend to experience longer periods of bed rest, more pronounced limitations in limb mobility, and insufficient participation in rehabilitation, making them more prone to an overall decline in muscle function. On top of these factors, if patients also have multiple comorbidities, their overall inflammation levels and nutritional risks may further increase, collectively influencing the development of stroke-related sarcopenia.

Therefore, stroke severity and the burden of comorbidity are not only clinical characteristics of the disease itself but also serve as key factors for the early identification of post-stroke sarcopenia risk. Clinically, stroke severity assessment should be integrated with comorbidity management to prioritize monitoring and early intervention for patients with high NIHSS scores and multiple comorbidities.

### 4.3 Treatment exposure: a comprehensive reflection of disease severity and a key component of clinical decision-making

Treatment-related factors play a significant role in stroke-related sarcopenia, as they reflect a patient’s disease severity, functional status, and nutritional risk. Based on these factors, clinicians can guide interventions for high-risk populations.

Existing studies suggest that the detection rate of stroke-related sarcopenia may increase with prolonged hospitalization, particularly among patients admitted to the ICU. Results from Wang et al. (28) indicate that prolonged hospitalization is statistically associated with stroke-related sarcopenia, with an OR of 1.27 (1.11–1.46); He et al. (25) found that patients with stroke-related sarcopenia had a hospital stay that was, on average, 0.4 days longer than those without sarcopenia, with a mean difference (MD) of 0.40 (0.20–0.59). This result suggests that a prolonged hospital stay not only reflects the severity of the condition but also indicates that patients experience prolonged bed rest, reduced physical activity, increased inflammatory burden, and inadequate nutritional intake. For patients in the ICU, tube feeding and other nutritional support methods are often medically necessary because of the severity of the stroke, the difficulty of swallowing, or the loss of consciousness. Such patients typically have a higher probability of developing pneumonia (49), which may be related to multiple factors including disease severity, increased risk of aspiration, feeding intolerance, and oral hygiene issues. These factors not only affect nutritional status but may also further impact muscle function by exacerbating the inflammatory response and reducing physical activity.

In addition to ICU patients, stroke patients in the recovery phase may also experience impaired eating ability and reduced willingness to eat due to oral health issues and swallowing dysfunction, leading them to prefer foods with a softer texture. Although this approach helps reduce the risk of aspiration to some extent, it may also increase the risk of inadequate nutritional intake, which in turn affects skeletal muscle mass; once stroke patients develop sarcopenia, it accelerates the functional decline of swallowing-related muscle groups, creating a vicious cycle of “swallowing dysfunction—malnutrition—sarcopenia.” Therefore, the management of associated factors should involve a comprehensive assessment that takes into account disease severity, nutritional support methods, and the patient’s functional status. Early intervention should include proper enteral feeding care to ensure oral hygiene and adequate nutritional intake, thereby reducing the risk of nutritional loss.

### 4.4 Behavioral environment, nutritional risk, and physical function: key entry points for clinical intervention

Behavioral environment, nutritional risk, and physical function are modifiable factors in stroke-related sarcopenia and serve as key entry points for clinical management. Active nutritional support promotes recovery from post-stroke sarcopenia and contributes to functional improvement during neurological recovery (50). However, most current research on nutritional supplements has focused on elderly individuals with sarcopenia. For patients with stroke-related sarcopenia, further investigation is needed to determine which nutritional strategy is most appropriate, taking into account the pathophysiological changes in stroke patients.

For patients with oral health issues and swallowing disorders, common feeding methods include nasogastric tubes, nasojejunal tubes, intermittent tube feeding, percutaneous endoscopic gastrostomy (PEG), and oral feeding (51). A meta-analysis involving 1,416 patients with post-stroke dysphagia compared percutaneous endoscopic gastrostomy (PEG) with nasogastric tube feeding and found that, compared with nasogastric tube feeding, PEG may be associated with a reduced risk of pneumonia, higher serum albumin levels, and lower overall mortality (52). However, this study included a small number of studies, had a limited sample size, and the timing and duration of different feeding methods were not standardized; therefore, this conclusion is subject to some bias. Future multicenter, large-scale studies are still needed to conduct a more in-depth comparison of different feeding methods in order to identify the most suitable nutritional support regimen for stroke patients, reduce the incidence of nutritional risks and other complications, and thereby lower the risk of sarcopenia in stroke patients.

In addition to nutritional risks, unhealthy behaviors such as smoking can also be addressed through early clinical intervention. Smoking may indirectly increase the likelihood of post-stroke sarcopenia by exacerbating oral health problems, swallowing difficulties, and chronic inflammatory states. Therefore, for the elderly population—especially those with a history of stroke—smoking cessation education should be strengthened to reduce the adverse effects of such behaviors on nutrition and muscle function.

Regarding exercise interventions, walking is a simple and accessible form of exercise for older adults, and walking speed is closely associated with blood lipid levels, particularly high-density lipoprotein (HDL) cholesterol and triglyceride levels (53). In addition to walking, resistance training combined with nutritional therapy may also help improve glycemic control (54). These findings suggest that exercise is not only relevant to sarcopenia management but may also simultaneously influence metabolic parameters such as blood glucose and lipid levels. Clinically, healthcare professionals should develop individualized exercise regimens—including type, frequency, and duration—for stroke patients based on an assessment of their clinical condition, functional status, and balance ability, and they should enhance rehabilitation outcomes through multidisciplinary collaboration.

### 4.5 Future research directions

Currently, there are 53 screening tools available to assess the risk of sarcopenia (55); among them, questionnaires such as the SARC-F are already widely used in clinical practice, but the validity of other methods—such as imaging studies and biochemical markers—remains to be further validated. Certain related factors can assist in identifying high-risk patients for post-stroke sarcopenia in clinical practice: for example, monitoring inflammatory markers such as high-sensitivity C-reactive protein and lymphocytes, as well as lipid profiles, during the clinical course can help in the early identification of high-risk patients. Inflammation accelerates muscle loss, and alleviating inflammation is directly correlated with patients’ activities of daily living (ADL) (56). However, it remains unclear whether these elevated inflammatory markers are caused by the stroke itself or are attributable to complications. This uncertainty may impact diagnosis and treatment (57). Furthermore, the sensitivity and accuracy of biochemical markers used to aid in the diagnosis of sarcopenia are susceptible to various factors, including environmental conditions and medication (55). Therefore, further research is needed to explore the role of biochemical markers in screening for stroke-related sarcopenia. As research has progressed, scientists have found that the phase angle is associated with malnutrition, length of hospital stay, physical function, inflammatory status, and sarcopenia (58). The phase angle is a key indicator derived from bioimpedance analysis (BIA) technology. Due to its non-invasive and convenient nature, it is commonly used in clinical settings to assess nutritional status and the risk of sarcopenia, and it can also assist in adjusting patients’ dietary intake and functional exercise regimens (33).

Among the studies included in this review, only one original study reported an association between reduced gut microbiota diversity and stroke-related sarcopenia (35). The gut microbiota is closely linked to human health and is regarded as the body’s “second brain” (59). In recent years, a growing number of studies have found that metabolites produced by the gut microbiota can regulate skeletal muscle protein synthesis through multiple pathways, leading to the proposal of the “gut-muscle axis” theory (60). Existing studies (61, 62) indicate that short-chain fatty acids are key metabolites of the gut microbiota. These short-chain fatty acids promote the expression of genes involved in muscle protein synthesis via the mTOR/IGF-1 signaling pathway; they enhance muscle mass and improve muscle cross-sectional area by increasing muscle glycogen levels and inhibiting histone deacetylases; and they also improve the metabolic efficiency of muscle fibers by increasing adenosine triphosphate (ATP) production. Conversely, dysbiosis of the gut microbiota may disrupt the secondary bile acid–FXR signaling pathway and skeletal muscle protein synthesis, potentially influencing the development of sarcopenia (63). Furthermore, short-chain fatty acids—metabolites of the gut microbiota—play a significant role in changes to muscle fibers in elderly patients with type 2 diabetes (64). These findings have important clinical implications for the screening and assessment of post-stroke sarcopenia and provide potential therapeutic targets for its treatment. In the future, further research is needed to explore whether the gut microbiota can serve as a screening marker for post-stroke sarcopenia and whether probiotic supplementation and fecal transplantation can aid in the recovery from stroke-related sarcopenia, as well as to elucidate the underlying mechanisms (65).

## 5 Limitations

This study has the following limitations: First, the majority of the original studies included in this review were conducted in Asia; 71.4% (20/28) of these studies used the AWGS as the diagnostic criterion for stroke-related sarcopenia, and most did not distinguish between different types of stroke. Due to differences in study populations and diagnostic criteria for sarcopenia, their representativeness is limited. Second, cross-sectional studies accounted for 62.5% (20/32) of the included studies, making it difficult to establish clear causal relationships between individual associated factors and sarcopenia. Further multicenter, large-sample cohort studies are needed to identify associated factors for stroke-related sarcopenia across different countries and populations, while also exploring the dose–response relationships between various associated factors and stroke-related sarcopenia, thereby providing a basis for clinical screening and intervention. Third, there was significant heterogeneity in the definitions of sarcopenia used in the studies included in this review: they ranged from formal diagnostic criteria such as AWGS, EWGSOP2, and FNIH to screening tools such as SARC-F. However, SARC-F is primarily used for screening for sarcopenia and is not equivalent to a diagnostic criterion. Therefore, the variability in definitions may be one of the key reasons for the wide range of sarcopenia prevalence rates (15.8–68.4%) among stroke patients and the inconsistent results regarding associated factors. Fourth, the vast majority of studies included in this review lacked pre-stroke baseline assessments, making it difficult to strictly distinguish between sarcopenia that developed after stroke and sarcopenia that existed before stroke but was only identified after the event. Therefore, prospective cohort studies are needed in the future to further clarify the association between stroke and sarcopenia.

## 6 Conclusion

This study identified 32 associated factors associated with stroke-related sarcopenia which interact through various pathways—including physiological, disease-related, therapeutic, and behavioral factors—to influence the development of sarcopenia in stroke patients. In clinical practice, healthcare professionals should conduct comprehensive assessments tailored to each patient’s specific circumstances and intervene early in modifiable factors, such as ensuring adequate nutritional intake and developing exercise regimens. They should also establish dynamic monitoring protocols, including regular measurements of phase angle, calf circumference, and relevant biochemical markers, to reduce the risk of sarcopenia and improve prognosis.
